# Supplementary material for: Determinants of timely administration of the birth dose of hepatitis B vaccine in Senegal in 2019: Secondary analysis of the demographic and health survey
Source: PLOS Glob Public Health. 2024 Aug 27;4(8):e0002734. doi: 10.1371/journal.pgph.0002734 (PMC11349090; doi:10.1371/journal.pgph.0002734)
Supplement: S1 Table — (DOCX) [file pgph.0002734.s001.docx]

S1 Table: Characteristics of participants, DHS, 2019, N=1130

| Characteristics | n (%) |
| --- | --- |
| Individual characteristics |  |
| Age of mother (years) |  |
| < 25 | 315 (27.9%) |
| [25 to 34] | 544 (48.1%) |
| ≥35 | 272 (24.0%) |
| Mother's education level |  |
| None | 679 (60.1%) |
| Primary | 233 (20.6%) |
| Secondary/tertiary | 219 (19.3%) |
| Marital status of mother |  |
| Unmarried | 59 (5.2%) |
| Married | 1,072 (94.8%) |
| Birth order |  |
| 1-2 | 505 (44.7%) |
| > 2 | 625 (55.3%) |
| Sex of the child |  |
| Female | 602 (53.2%) |
| Male | 528 (46.8%) |
| Contextual characteristics |  |
| Area of residence |  |
| West | 349 (30.9%) |
| Center | 397 (35.2%) |
| South | 198 (17.5%) |
| North | 186 (16.4%) |
| Type of residence |  |
| Rural | 702 (62.1%) |
| Urban | 428 (37.9%) |
| Household size |  |
| ≤ 6 persons | 207 (18.3%) |
| > 6 persons | 923 (81.7%) |
| Sex of household head |  |
| Female | 301 (26.6%) |
| Male | 829 (73.4%) |
| Wealth quintile |  |
| The poorest/poorer | 490 (43.3%) |
| Middle | 215 (19.0%) |
| Richer/richest | 426 (37.7%) |
| Father's education level |  |
| None | 799 (74.5%) |
| Primary | 87 (8.1%) |
| Secondary/tertiary | 187 (17.4%) |
| Missing | 57 |
| TT injection |  |
| Yes | 997 (91.4%) |
| No | 94 (8.6%) |
| Missing | 40 |
| Number of ANC |  |
| <4 | 497 (45.6%) |
| ≥4 | 593 (54.4%) |
| Missing | 40 |
| Place of birth |  |
| Health facility | 933 (82.6%) |
| Outside health facility | 197 (17.4) |
| Mode of delivery |  |
| Cesarean section | 50 (4.4%) |
| Vaginal delivery | 1080 (95.6%) |
| Checking the child's health before leaving the health facility |  |
| No/don't know | 6 (0.7%) |
| Yes | 894 (99.3%) |
| Missing | 230 |
| Possession of HBR |  |
| No | 25 (2.3%) |
| No longer has a HBR | 110 (9.8%) |
| Yes, not seen | 53 (4.7%) |
| Yes, seen | 941(83.3%) |

TT: Tetanus Toxoid; ANC: Antenatal Care; HBR: Health Birth Record
