# Supplementary material for: Determinants of timely administration of the birth dose of hepatitis B vaccine in Senegal in 2019: Secondary analysis of the demographic and health survey
Source: PLOS Glob Public Health. 2024 Aug 27;4(8):e0002734. doi: 10.1371/journal.pgph.0002734 (PMC11349090; doi:10.1371/journal.pgph.0002734)
Supplement: S2 Table — (DOCX) [file pgph.0002734.s002.docx]

S2 Table: Bivariate analysis of timely HepB0 vaccination, DHS, 2019, N=747.

| Characteristics | | HepB0≤24 hours,  N = 487 | HepB0>24 hours  N = 260 | OR [95% CI] | p-value |
| --- | --- | --- | --- | --- | --- |
| Individual characteristics | |  |  |  |  |
| Age of the mother | |  |  |  | 0.016 |
| <25 | | 142 (63.3%) | 82 (36.7%) | 1 |  |
| [25 to 34] | | 219 (61.5%) | 137(38.5%) | 0.93 [0.61-1.41] |  |
| ≥35 | | 126 (75.6%) | 41 (24.4%) | 1.79 [1.16-2.77] |  |
| Mother's education level | |  |  |  | <0.001 |
| None | | 248 (57.6%) | 183(42.4%) | 1 |  |
| Primary | | 139 (81.2%) | 32 (18.8%) | 3.18 [1.94-5.20] |  |
| Secondary/tertiary | | 100 (69.0%) | 45 (31.0%) | 1.64 [1.04-2.58] |  |
| Marital status of the mother | |  |  |  | 0.067 |
| Not married | | 29 (78.9%) | 8 (21.1%) | 1 |  |
| Married | | 458 (64.5%) | 252(35.5%) | 0.48 [0.22-1.07] |  |
| Birth order | |  |  |  | 0.780 |
| 1-2 | | 231 (65.9%) | 120(34.1%) | 1 |  |
| > 2 | | 256 (64.6%) | 140(35.4%) | 0.95 [0.65-1.38] |  |
| Sex of the child | |  |  |  | 0.326 |
| Female | | 266 (67.1%) | 130(32.9%) | 1 |  |
| Male | | 221 (63.1%) | 129(36.9%) | 0.84 [0.59-1.19] |  |
| Contextual characteristics | |  |  |  |  |
| Area of residence | |  |  |  | <0.001 |
| West | | 210 (85.8%) | 35 (14.2%) | 1 |  |
| Center | | 131(51.9%) | 121(48.1%) | 0.18 [0.08-0.38] |  |
| South | | 88 (66.0%) | 45 (34.0%) | 0.32 [0.16-0.67] |  |
| North | | 59 (50.0%) | 59 (50.0%) | 0.17 [0.08-0.36] |  |
| Type of residence | |  |  |  | <0.001 |
| Rural | | 258 (58.0%) | 187(42.0%) | 1 |  |
| Urban | | 229 (75.9%) | 73 (24.1%) | 2.29 [1.46-3.58] |  |
| Household size | |  |  |  | 0.125 |
| ≤6 persons | | 90 (72.5%) | 34 (27.5%) | 1 |  |
| > 6 persons | | 397 (63.7%) | 226(36.3%) | 0.67 [0.39-1.12] |  |
| Wealth quintile | |  |  |  | <0.001 |
| Poor | | 155 (52.8%) | 138(47.2%) | 1 |  |
| Middle | | 94 (65.2%) | 50 (34.8%) | 1.67 [0.98-2.84] |  |
| Rich | | 239 (76.9%) | 72 (23.1%) | 2.98 [1.87-4.76] |  |
| Sex of household head | |  |  |  | 0.891 |
| Female | | 127 (64.7%) | 70 (35.3%) | 1 |  |
| Male | | 359 (65.4%) | 190(34.6%) | 1.03 [0.66-1.60] |  |
| Father's education level | |  |  |  | 0.143 |
| None | | 316 (61.5%) | 198(38.5%) | 1 |  |
| Primary | | 52 (76.2%) | 16 (23.8%) | 2.01 [0.98-3.89] |  |
| Secondary/tertiary | | 92 (70.9%) | 38 (29.1%) | 1.53 [0.73-3.18] |  |
| Missing | | 27 | 8 |  |  |
| TT injection | |  |  |  | 0.064 |
| No | | 30 (50.8%) | 29 (49.2%) | 1 |  |
| Yes | | 438 (66.2%) | 224(33.8%) | 1.90 [0.95-3.78] |  |
| Missing | | 18 | 7 |  |  |
| Number of ANC | |  |  |  | <0.001 |
| <4 | | 181 (55.9%) | 143(44.1%) | 1 |  |
| ≥4 | | 287 (72.3%) | 110(27.7%) | 2.06 [1.41-3.03] |  |
| Missing | | 18 | 7 |  |  |
| Place of birth | |  |  |  | <0.001 |
| Outside health facility | | 34 (33.8%) | 66 (66.2%) | 1 |  |
| Health facility | | 453 (70.1%) | 194(29.9%) | 4.59 [2.71-7.75] |  |
| Mode of delivery | |  |  |  | 0.397 |
| Vaginal delivery | | 461 (64.8%) | 250(35.2%) | 1 |  |
| Cesarean section | | 26 (73.0%) | 10 (27.0%) | 1.47 [0.60-3.58] |  |
| Checking the child's health before leaving the health facility | |  |  |  | 0.802 |
| No/don't know | | 2 (76.4%) | 1 (23.6%) | 1 |  |
| Yes | | 435 (69.8%) | 189(30.2%) | 0.71 [0.05-10.4] |  |
| Missing | | 50 | 71 |  |  |
| Access to newspaper/magazine | |  |  |  | 0.013 |
| No | | 377 (62.0%) | 232(38.0%) | 1 |  |
| Yes | | 109 (79.6%) | 28 (20.4%) | 2.40 [1.19-4.86] |  |
| Radio access | |  |  |  | 0.251 |
| No | | 89 (59.4%) | 61 (40.6%) | 1 |  |
| Yes | | 398 (66.7%) | 199(33.3%) | 1.36 [0.80-2.33] |  |
| Access to television | |  |  |  | 0.001 |
| No | | 78 (50.9%) | 76 (49.1%) | 1 |  |
| Yes | | 408 (68.9%) | 184(31.1%) | 2.14 [1.35-3.39] |  |
| Mobile phone access | |  |  |  | <0.001 |
| No | | 132 (54.0%) | 113(46.0%) | 1 |  |
| Yes | | 355 (70.7%) | 147(29.3%) | 2.05 [1.41-2.99] |  |
| Internet access | |  |  |  | <0.001 |
| No | | 245 (57.7%) | 180(42.3%) | 1 |  |
| Yes | | 242 (75.1%) | 80 (24.9%) | 2.21 [1.40-3.49] |  |
|  | ^1^ n (%) | | | | |
|  | ^2^ Chi² test with the second order correction of Rao & Scott | | | | |

TT: Tetanus Toxoid; ANC: Antenatal Care
