# Supplementary material for: Determinants of timely administration of the birth dose of hepatitis B vaccine in Senegal in 2019: Secondary analysis of the demographic and health survey
Source: PLOS Glob Public Health. 2024 Aug 27;4(8):e0002734. doi: 10.1371/journal.pgph.0002734 (PMC11349090; doi:10.1371/journal.pgph.0002734)
Supplement: S3 Table — (DOCX) [file pgph.0002734.s003.docx]

S3 Table: Multivariate analysis of significant determinants of timely HepB0 vaccination, DHS, 2019, N=747

| Features | aOR [95%CI] |
| --- | --- |
| Individual characteristics |  |
| Age of mother: ≥ 35 years versus < 35 years | 2.03; [1.29 – 3.20] |
| Age of mother: [25 to 34] versus < 35 years | 0.95; [0.61 – 1.49] |
| Mother's education level: primary versus none | 1.94; [1.13 – 3.35] |
| Mother's education level: Secondary/tertiary versus none | 0.77; [0.37 – 1.62] |
| Contextual characteristics |  |
| Area of residence: Center versus West | 0.22; [0.11 – 0.44] |
| Area of residence: North versus West | 0.18; [0.08 – 0.40] |
| Area of residence: South versus West | 0.57; [0.28 – 1.13] |
| Number of ANC: ≥ 4 versus <4 | 1.74; [1.12 – 2.69] |
| Place of delivery: health facility versus outside health facility | 3.42; [1.90 – 6.15] |

ANC: Antenatal Care
