# Supplementary material for: Determinants of timely administration of the birth dose of hepatitis B vaccine in Senegal in 2019: Secondary analysis of the demographic and health survey
Source: PLOS Glob Public Health. 2024 Aug 27;4(8):e0002734. doi: 10.1371/journal.pgph.0002734 (PMC11349090; doi:10.1371/journal.pgph.0002734)
Supplement: Attachment — Submitted filename: Secondary analysis of the demographic and health survey.docx [file pgph.0002734.s004.docx]

Re: Determinants of timely administration of the birth dose of hepatitis B vaccine in

Senegal in 2019: Secondary analysis of the demographic and health survey

Thanks for allowing me to revise this important topic on hepatitis B birth dose, a secondary analysis of DHS 2019 in Senegal

General comments: Though generally well written, there a few comments for authors

Abstract: “In total, 1130 children were included. Among them, 48.1% were born to mothers aged 25 to 34, 46.8% were male and 82.6% were born in health facilities. The average time between birth and HepB0 administration was 13.1±46.6 days. Among 747 children vaccinated, only 65.2” - Unclear, you included 1130, 747 were vaccinated? you are interested in timeliness ?

“13.1±46.6 days.” Present as median with IQR, add range.

Introduction: “A recent study estimates that 296 million people will be chronically infected and 820,000 will die worldwide in 2019 [1]- Kindly recast? we are 2024!!! If it is projected, recast to reflect past event on projection

Methods: Kindly include sample size estimation?

“Model adequacy was analyzed using the Hosmer-Lemeshow test.” No information on this in the results?

Results: Participant characteristics-include n before the percentage.

Vaccination coverage: present the measure of central tendency with median plus IQR, add range as well.

“Among them, 65.2% (487/747) were vaccinated on time, i.e. within 24 hours of birth” why is this not the actual sample size?

Multivariate analysis-change to binary logistic regression

Multivariate is actually multivariable analysis-your outcome is still binary-the only thing is that you entered multiple variables!!!

Table 3: There are quite a number of variables that were significant on 2 by 2 in Table 2, they were not included in Table 3?

Vaccination coverage: It will be nice to compare your data with a large country with similar challenges in the immunization coverage -see Assessment of the Timely Administration of Birth Dose Vaccines in Northern Nigeria and Associated Factors (Ann Glob Health. 2022; 88(1): 60.Published online 2022 Jul 26. (doi: 10.5334/aogh.3743)
