# Supplementary material for: Determinants of timely administration of the birth dose of hepatitis B vaccine in Senegal in 2019: Secondary analysis of the demographic and health survey
Source: PLOS Glob Public Health. 2024 Aug 27;4(8):e0002734. doi: 10.1371/journal.pgph.0002734 (PMC11349090; doi:10.1371/journal.pgph.0002734)
Supplement: Attachment — Submitted filename: Response to Reviewers.docx [file pgph.0002734.s006.docx]

Re: Determinants of timely administration of the birth dose of hepatitis B vaccine in

Senegal in 2019: Secondary analysis of the demographic and health survey

Thanks for allowing me to revise this important topic on hepatitis B birth dose, a secondary analysis of DHS 2019 in Senegal

General comments: Though generally well written, there a few comments for authors

*** Thank you very much for appreciating the article. We will take your suggestions into account to improve the article.

Abstract: “In total, 1130 children were included. Among them, 48.1% were born to mothers aged 25 to 34, 46.8% were male and 82.6% were born in health facilities. The average time between birth and HepB0 administration was 13.1±46.6 days. Among 747 children vaccinated, only 65.2” - Unclear, you included 1130, 747 were vaccinated? you are interested in timeliness ?

*** Thank you very much for the glance. The 747 concern children vaccinated for any length of time. For the remaining "65.2%", this concerns the periodicity according to the WHO recommendation (within 24 hours) among those vaccinated and for whom we had the period.

“13.1±46.6 days.” Present as median with IQR, add range.

*** It's integrated on page 2.

Introduction: “A recent study estimates that 296 million people will be chronically infected and 820,000 will die worldwide in 2019 [1]- Kindly recast? we are 2024!!! If it is projected, recast to reflect past event on projection.

*** This is not a projection. It's an incorrect conjugation that we've corrected (page 3).

Methods:

Kindly include sample size estimation?

*** Thanks for the comment. We have integrated the sampling estimation method..

“Model adequacy was analyzed using the Hosmer-Lemeshow test.” No information on this in the results?

*** We have removed it because it is not searchable with weighted data in R.

Results:

Participant characteristics-include n before the percentage.

*** However, in the tables, we have integrated the n's before the percentages. But this is done in brackets in the text.

Vaccination coverage: present the measure of central tendency with median plus IQR, add range as well.

*** It's done on page 12.

“Among them, 65.2% (487/747) were vaccinated on time, i.e. within 24 hours of birth” why is this not the actual sample size?

*** Thank you very much for your comment. As said above, it concerns the periodicity according to the WHO recommendation (within 24h) among those who are vaccinated and for whom had the period as specified by the sentence just before.

Multivariate analysis-change to binary logistic regression

*** Done

Multivariate is actually multivariable analysis-your outcome is still binary-the only thing is that you entered multiple variables!!!

*** Done

Table 3: There are quite a number of variables that were significant on 2 by 2 in Table 2, they were not included in Table 3?

*** Thanks for the comment. Yes it's because we applied a top-down step-by-step approach as specified in the methodology to have the most parsimonious model.

Vaccination coverage: It will be nice to compare your data with a large country with similar challenges in the immunization coverage -see Assessment of the Timely Administration of Birth Dose Vaccines in Northern Nigeria and Associated Factors (Ann Glob Health. 2022; 88(1): 60.Published online 2022 Jul 26. (doi: 10.5334/aogh.3743)

*** Thank you very much for suggesting this relevant study. We have included it in the comparison (Page 20).
