# Supplementary material for: Determinants of timely administration of the birth dose of hepatitis B vaccine in Senegal in 2019: Secondary analysis of the demographic and health survey
Source: PLOS Glob Public Health. 2024 Aug 27;4(8):e0002734. doi: 10.1371/journal.pgph.0002734 (PMC11349090; doi:10.1371/journal.pgph.0002734)
Supplement: Attachment — Submitted filename: Response to reviewers..docx [file pgph.0002734.s007.docx]

This is quite an important topic. Prevention of vertical transmission of hepatitis B needs to be improved in SSA. The authors present a secondary analysis of DHS 2019 Senegal data to assess the timeliness of the Hepatitis B vaccine and find its correlates. However, there are important technical issues in the analysis.

1. Was the complex survey sampling addressed in this analysis? The current statistical section says nothing about this. As the reviewer rightly points out there please provide more details on the statistical procedures used here.

*** Thank you very much for your comment. The data has indeed been weighted and we have taken into account the complex sampling plan. Sentences have been added to this effect on page 7.

2. Table 2 uses bivariate p-values and no measure of association such as OR or RR or RD. Please put the measure of association and its 95%CI.
Please try to put them into single table 1 and table 2.

*** Thank you very much for your comment. Tables 1 and 2 do not have the same Ns (the reason for this is explained in the first phase of revision). However, we have added the measures of association (crude OR) and the 95% CI to table 2 (bivariate analysis).

3. Table 3: It does not make sense to include the same variable in one categorization in table 2 then in table 3 use another. For example, education primary versus none. Where are are other categories?

*** Thank you very much for your comment. In fact, the same categories have been included. Only those that are significant compared to the reference are left in the table. However, in this case, we have now included the other modality as well as the other variables concerned so as not to create confusion.

4. Please reorganize the discussion section. Move the strengths and limitations of the study to the end of discussion section.

*** We've moved it to the end. Thank you for your time.
