# Supplementary material for: Determinants of timely administration of the birth dose of hepatitis B vaccine in Senegal in 2019: Secondary analysis of the demographic and health survey
Source: PLOS Glob Public Health. 2024 Aug 27;4(8):e0002734. doi: 10.1371/journal.pgph.0002734 (PMC11349090; doi:10.1371/journal.pgph.0002734)
Supplement: Attachment — Submitted filename: Response to reviewers.docx [file pgph.0002734.s009.docx]

**Please address the following items.**

Please remove the tables from the main text as they are bulky and submit them as supplements

*** We will remove the tables from the main text as requested and submit them as additional documents.

Some categories in table 2 are not clear: What does "checking the child's health before leaving the health facility" represent?

*** The category “checking the child's health before leaving the health facility" corresponds to a check on the child's health by a health professional before the mother and newborn leave the facility after delivery.

(Table 2) Please use standard medical terminology for mode of delivery

*** It’s done

(Table 2) Describe abbreviations at the bottom of the table

*** It’s done

(Table 2) How is household lead determined?

*** The head of household is determined during the demographic survey. This is generally the person recognized as such by the other members.

(Table 2) Quantify the wealth quintiles

*** We cannot quantify quintiles. This is a secondary data analysis, and the variable was given as is by the Agence Nationale de la Statistique et de la Démographie. The factor calculations used to produce the quintiles are not included in the database.

(Table 2) Elaborate “area of residence” – are those the names of regions?

*** These are not names of regions. It's more a breakdown of regions by axis.

Include missing comparison categories into the multivariate analysis discussion in table 3

***We didn't understand enough. However, we usually focus more on our interpretation of significant multivariate results in the results and discussions.
