# Supplementary material for: Determinants of timely administration of the birth dose of hepatitis B vaccine in Senegal in 2019: Secondary analysis of the demographic and health survey
Source: PLOS Glob Public Health. 2024 Aug 27;4(8):e0002734. doi: 10.1371/journal.pgph.0002734 (PMC11349090; doi:10.1371/journal.pgph.0002734)
Supplement: Attachment — Submitted filename: Response to reviewers.docx [file pgph.0002734.s010.docx]

**Please address the following items.**

Please remove wealth quintiles if they cannot be quantified. Or describe this gap as a limitation for analysis by clearly outlining why the variable cannot be quantified.

*** Thank for the comment. In this sense, we have added 2 sentences in the part “limit”.

Please use formal region names instead of crude axis directions, which are subjective for analysis

*** Thank you for the relevant comment. The use of geographic axes instead of formal region names was determined by the National Agency for Statistics and Demography (ANSD) of Senegal. These axes materialize the representativeness of the sample, which the region names alone do not allow. We therefore recommend keeping this terminology as defined by the ANSD for the analysis of the DHS data (look page 20 (and all tables of document : <https://www.dhsprogram.com/pubs/pdf/FR368/FR368.pdf>).

If you have listed ONLY significant multivariate analysis in table 3, the title should say so

*** It’s done
